# Modified Retromandibular Approach for the Management of Condylar Fractures of the Mandible

**DOI:** 10.7759/cureus.27697

**Published:** 2022-08-05

**Authors:** Swetha V Bhat, Vinod K Krishna, Senthilnathan Periasamy, Santhosh P Kumar, Murugesan Krishnan

**Affiliations:** 1 Oral and Maxillofacial Surgery, Saveetha Dental College and Hospital, Chennai, IND

**Keywords:** innovative technique, dental occlusion, temporomandibular joint, mandibular fractures, retromandibular approach, condylar fractures, diacapitular fracture

## Abstract

Condylar fractures of the mandible have been of particular interest to surgeons for decades, as there is a big debate regarding the management option: conservative versus surgical modality. Conservative treatment is the preferred treatment modality for condylar head (diacapitular) fractures. Currently, surgical modality is favored and surgeons are opting for open reduction and internal fixation for diacapitular fractures as it reestablishes the anatomical position of the fragments and disc, and permits immediate functional mobility of the jaw, thereby reducing the chances of occurrence of temporomandibular joint ankylosis. This case series enumerates the pros and cons of open reduction and internal fixation of condylar fractures of the mandible occurring at various levels using a modified retromandibular approach and highlights that this can be considered as one of the treatment options for condylar fractures.

## Introduction

According to literature, mandibular fractures most frequently occur at the condyle, with incidence ranging from 21 to 49 percent [1]. The fixation of fractures of the mandibular condyle head, neck, and base has been a long-standing debatable topic in oral and maxillofacial surgery. Opinions range from the belief that all or nearly all displaced condylar fractures should be treated surgically to the belief that almost no condylar fractures require surgery [2]. Because of the technical difficulties in exposure and fixation as well as the risk of facial nerve injury, these fractures are usually treated conservatively. Although good initial clinical results are often achieved with conservative treatment, the occurrence of late sequelae like temporomandibular joint ankylosis, condylar necrosis, suppression of mandibular growth, and occlusal abnormalities have been described in the literature [3]. Unlike conservative treatment, open surgery can result in a speedy return of normal occlusion and jaw movement [4].

Conservative treatment of condylar head (diacapitular) fractures can result in extensive condylar deformation, reduction in height of the mandibular ramus, disc displacement, dysfunctional complaints (such as limitation of mandibular mobility, crepitation, and lateral deviation during mouth opening), temporomandibular joint (TMJ) ankylosis, and occlusal discrepancies [3]. Although closed reduction is an option for high condylar fractures, open reduction and internal fixation are preferred considering the advantages that include the restoration of the anatomical position of the fragments and disc, functional mobility of the jaw, and reducing the chances of occurrence of TMJ ankylosis [5,6]. Techniques that have been put forth to reduce as well as fix condylar head fractures are standard bone screws, resorbable screws, resorbable pins, and cannulated lag screws [7].

Computed Tomography (CT) scans have proved critical in determining the proper identification and prognosis of condylar head, neck, and base fractures, allowing for precise treatment planning. CT scans are the gold standard approach for diagnosing and classifying diacapitular fractures as they demonstrate the precise location of the fracture, the size, and orientation of the fragment, and, most substantially, how the ramus stump, fracture segment, and glenoid fossa interact with one another [8]. Diacapitular fractures in which the ramus stump dislocates laterally out of the glenoid fossa are indeed unquestionable absolute criteria for surgical treatment [1].

The most common approaches opted by surgeons for open reduction and internal fixation of diacapitular fractures include preauricular and retromandibular approaches. The preauricular approach provides limited access to the fracture site and complications of this approach include facial nerve paralysis, scar, and occurrence of Frey syndrome [9,10]. Hence we adopted a modified retromandibular approach to overcome the disadvantages of the preauricular approach. This article enumerates the outcomes of three cases of condylar fractures of the mandible (a condylar head, a condylar neck, and a subcondylar fracture) which were surgically treated with a modified retromandibular approach. 

## Case presentation

Case 1

**Figure 1:**
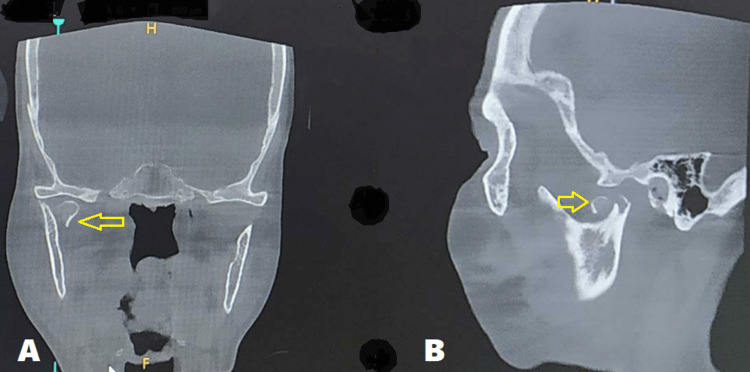
CT scan images CT scan showing medially displaced right condylar head fracture (arrows) in A) Coronal section B) Sagittal section

A 70-year-old male patient presented to the Oral and Maxillofacial Surgery department with a history of an automobile accident. The patient had a painful swelling over the right ear region, with limited mouth opening, deranged occlusion, and deviation of the mandible during the opening and closing of the jaws. Speech and function of the jaws were affected and the patient did not have any significant medical history. CT scan showed a medially displaced right condylar head (diacapitular) fracture (Figure 1). A surgical condylectomy procedure was planned for the patient as the fracture was three weeks old.

**Figure 2:**
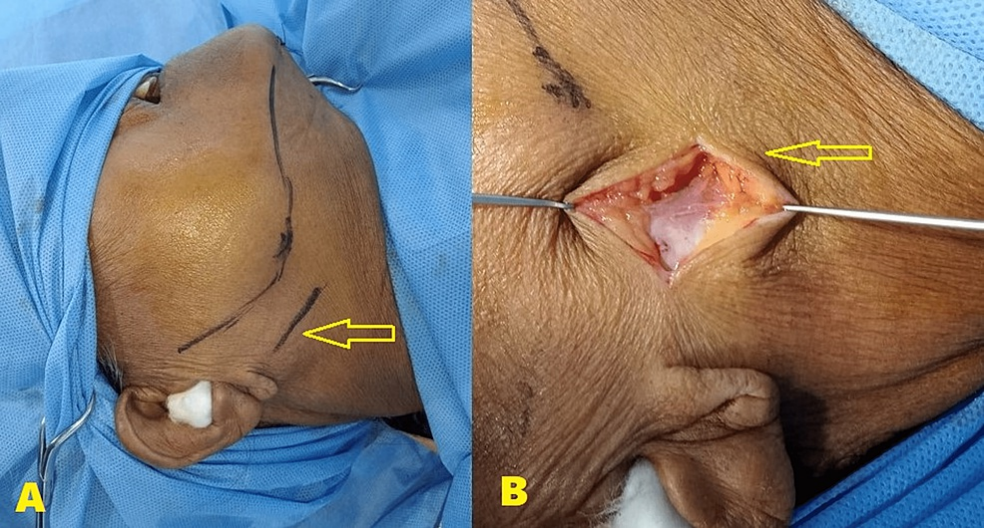
Preoperative and intraoperative images A) Markings for the retromandibular incision made parallel to the posterior border of the mandible (arrow) B) Incision and dissection of the scant platysma (arrow)

Under general anesthesia, arch bars were placed, intermaxillary fixation was done and good intraoperative occlusion was achieved. Markings were made and a 3-cm incision was done at 2 cm beyond the posterior border of the jaw and 0.5 cm below the ear lobe for the retromandibular approach. The incision was vertical and parallel to the mandibular posterior border and it extended to the level of the sparse platysma (Figure 2). The platysma muscle was incised in the same plane as the skin incision, and the parotid capsule and superficial musculoaponeurotic system (SMAS) were incised. Blunt dissection was performed below the tail of the parotid gland in an anteromedial orientation, following the facial nerve's predicted route at the posterior border of the mandible.

**Figure 3:**
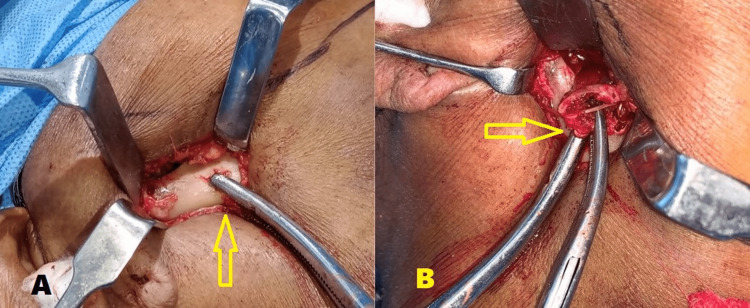
Intraoperative images A) Dissection of the pterygomasseteric sling and identification of the fracture segment (arrow) B) Retrieval of the fractured condylar segment (arrow)

The pterygomasseteric muscle sling was dissected towards the gonion until adequate access was obtained to the fracture segments (Figure 3). This was done to facilitate the retraction of the coronoid notch downwards which provided access to the fractured ends. The condylar head was identified and excised.

**Figure 4:**
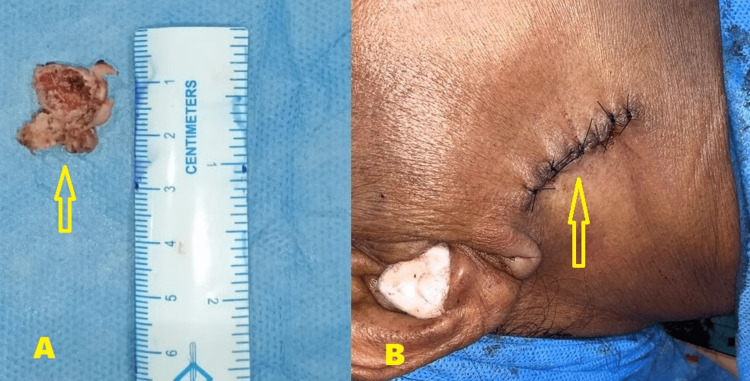
Postoperative images A) Excised right condylar head (arrow) B) Layered closure of the surgical wound (arrow)

**Figure 5:**
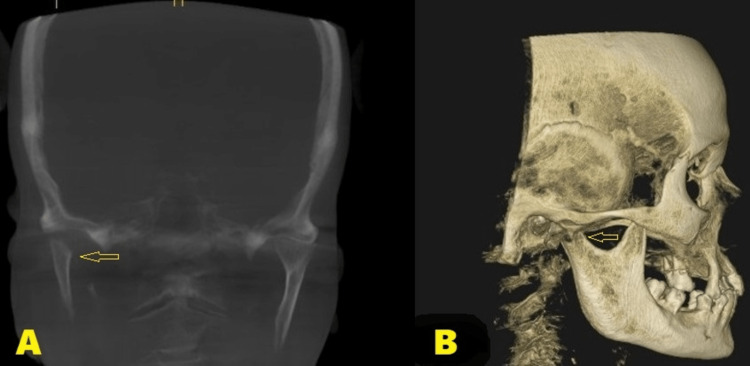
Postoperative CBCT scan Postoperative cone beam computed tomography (CBCT) scan showing good wound healing (arrows) in A) Coronal section B) Three-dimensional sagittal view

The skin was undermined and the closure was done in layers to avoid any salivary fistula formation (Figure 4). The patient had no postoperative complications associated with occlusion, mouth opening, mandibular range of motion, or facial nerve paralysis and had a good postoperative mouth opening in the normal range. A six-month postoperative cone beam computed tomography (CBCT) scan showed good wound healing and an absence of the right medial pole of the condyle (Figure 5), thus confirming the usefulness of the modified retromandibular approach in accessing condylar head (diacapitular) fractures of the mandible.

Case 2

**Figure 6:**
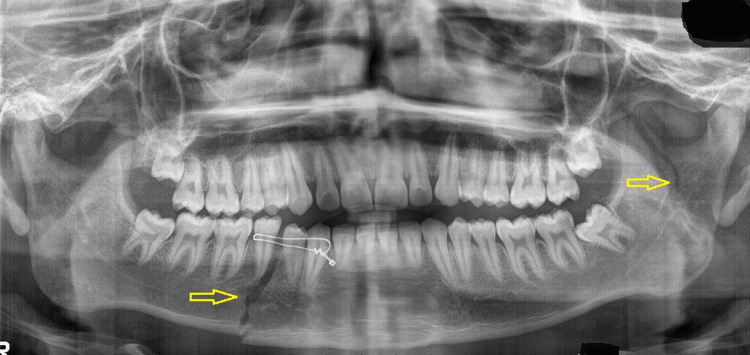
OPG image obtained at presentation Orthopantomogram (OPG) revealing a right parasymphysis and left subcondylar fracture (arrows)

An 18-year-old male patient presented to the Oral and Maxillofacial Surgery department with a history of an automobile accident. The patient had a painful swelling over the left ear region, with limited mouth opening and deviation of the mandible. Speech and function of the jaws were affected and the patient did not have any significant medical history. An orthopantomogram (OPG) revealed a right parasymphysis and left subcondylar fracture (Figure 6).

**Figure 7:**
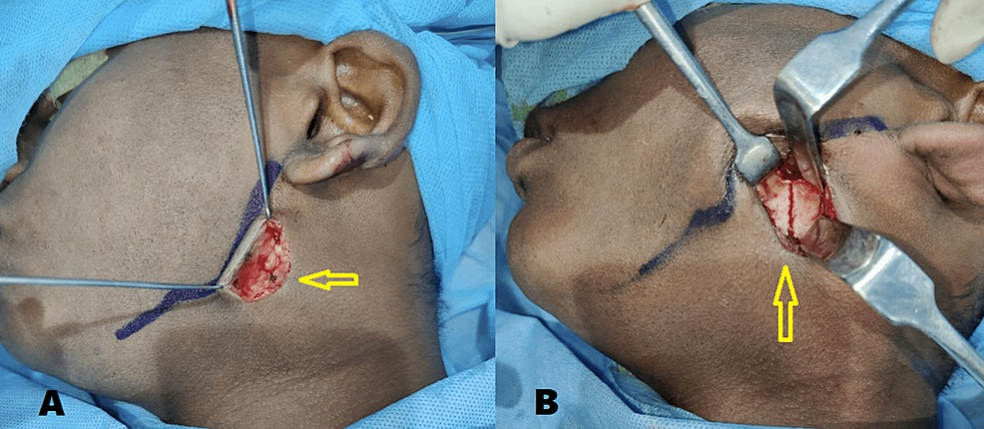
Intraoperative images A) Modified retromandibular approach (arrow) B) Exposure and reduction of the fractured condylar segment (arrow)

**Figure 8:**
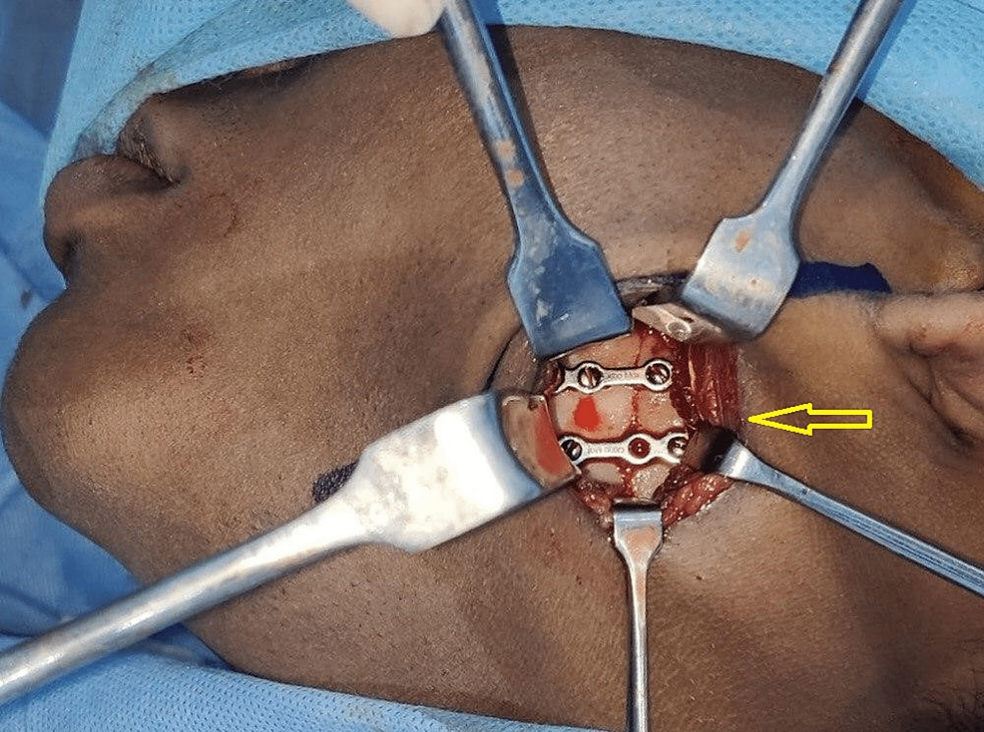
Fixation of the left subcondylar fracture (arrow)

Under general anesthesia, arch bars were placed, intermaxillary fixation was done and good intraoperative occlusion was achieved. Through the modified retromandibular approach, the fractured fragments were exposed, reduced (Figure 7), and fixed using two 2-mm stainless steel mini plates and screws (Figure 8). The wound was properly closed in layers to avoid salivary fistula formation.

**Figure 9:**
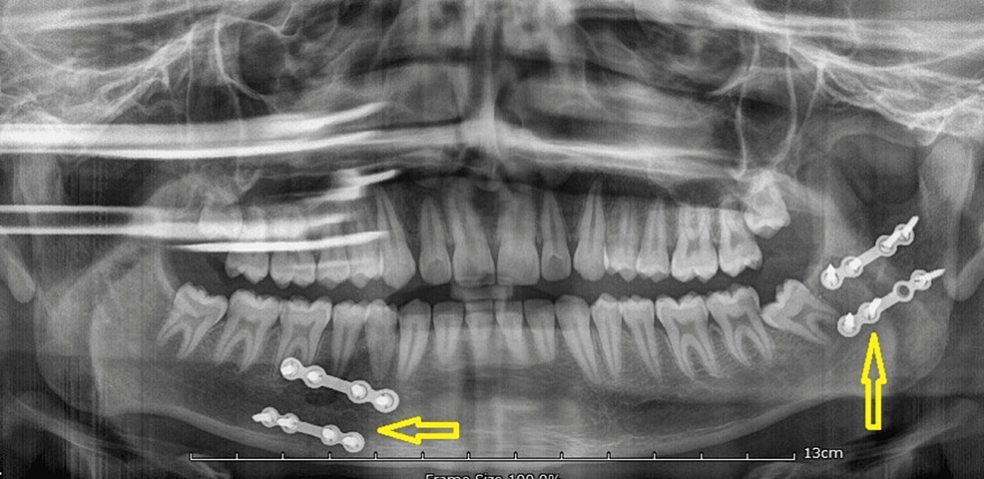
Postoperative OPG image Postoperative orthopantomogram (OPG) showing good wound healing (arrows)

The patient had no postoperative complications associated with occlusion, mouth opening, mandibular range of motion, or facial nerve paralysis. OPG during six months postoperative review showed a successful reduction and healing of the left sub-condylar and right parasymphysis fracture (Figure 9).

Case 3

**Figure 10:**
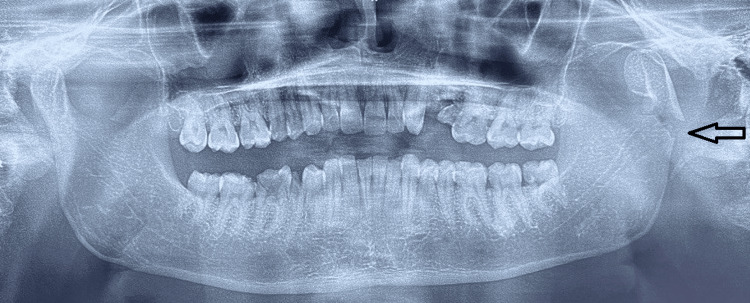
OPG revealing a left condylar neck fracture OPG: Orthopantomogram The arrow shows the left condylar neck fracture

A 30-year-old male patient presented to the Oral and Maxillofacial Surgery department with a history of an automobile accident. The patient had a painful swelling over the left ear region, with limited mouth opening and deviation of the mandible. The patient did not have any significant medical history. An OPG revealed a left condylar neck fracture (Figure 10).

**Figure 11:**
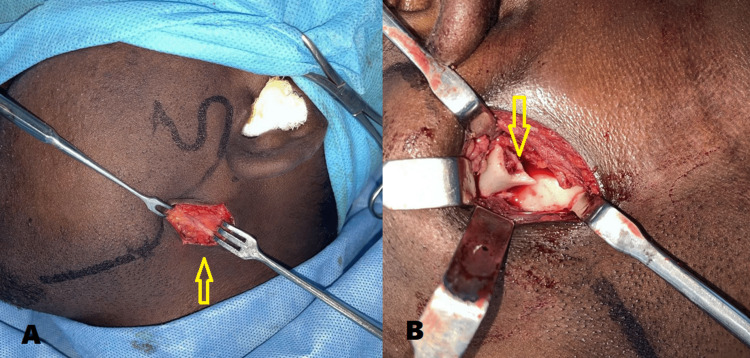
Intraoperative images A) Modified retromandibular approach (arrow) B) Exposure and retrieval of the fractured condylar segment (arrow)

**Figure 12:**
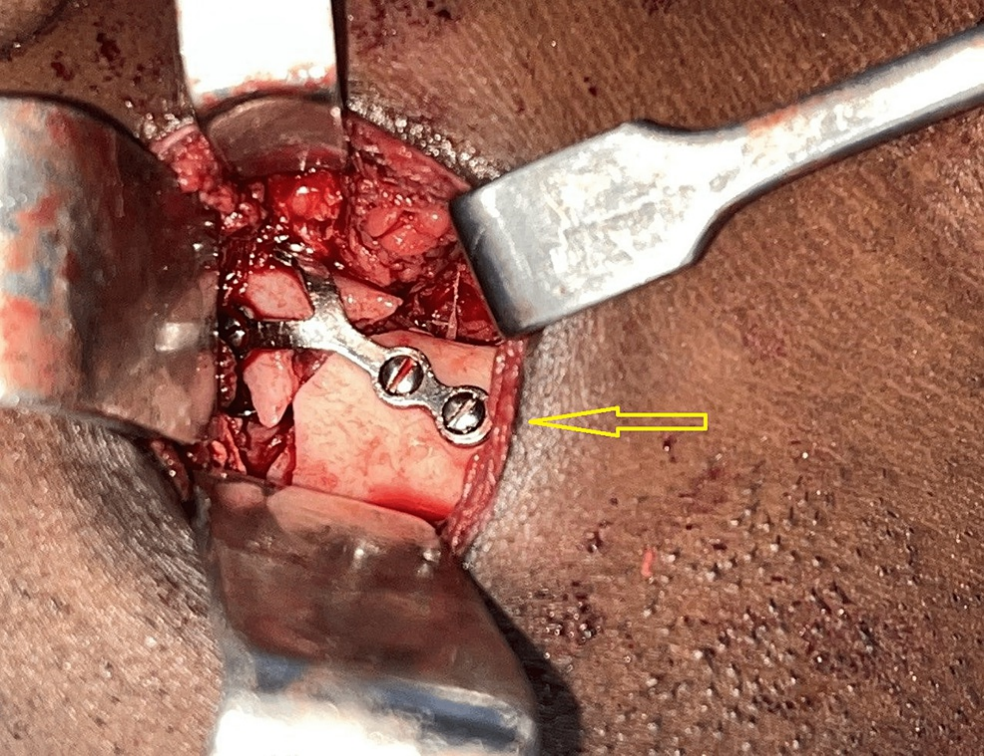
Extracorporeal fixation of the left condylar neck fracture Extracorporeal fixation of the left condylar neck fracture in the anatomically reduced position (arrow)

**Figure 13:**
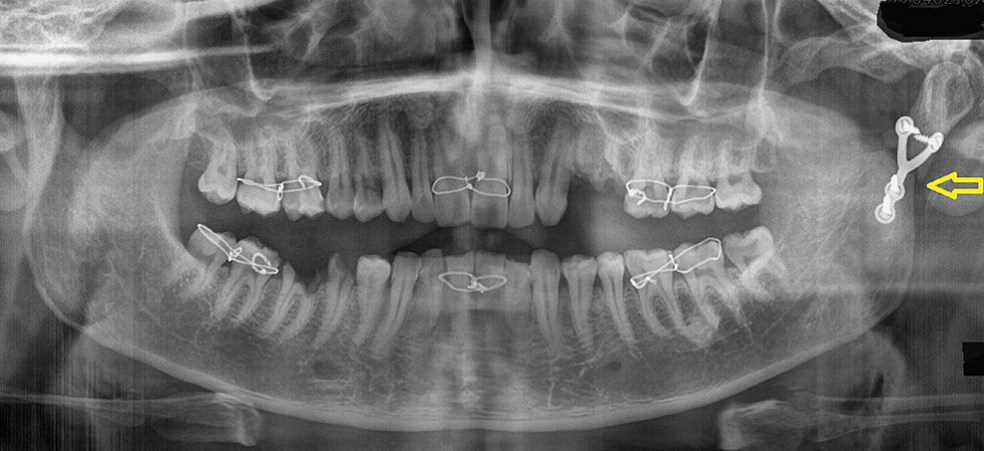
Postoperative OPG image OPG: Orthopantomogram Postoperative OPG showing good wound healing (arrow)

Under general anesthesia, arch bars were placed, intermaxillary fixation was done and good intraoperative occlusion was achieved. Through the modified retromandibular approach, the fractured fragments were exposed and the condylar fragment was retrieved (Figure 11). Extracorporeal fixation of the fractured condylar segment was done with proper anatomical reduction using a 2-mm titanium Y-plate and screws (Figure 12). The wound was properly closed in layers to avoid salivary fistula formation. The patient had no postoperative complications associated with occlusion, mouth opening, mandibular range of motion, or facial nerve paralysis and exhibited an inconspicuous scar. OPG during six months postoperative review showed a successful reduction and healing of the left condylar neck fracture (Figure 13).

## Discussion

Fractures of the condylar head have always been a controversial topic to be discussed due to the debatable results following a technically challenging open reduction and fixation versus conservative treatment. Children or individuals whose condylar head is comminuted are also advised to undergo conservative treatment [11-13]. The surgeon can determine whether open surgery is indicated for treating the fracture by using three-dimensional computed tomography (3D-CT) scan with computerized simulation. Condylar head fracture planning and treatment have benefited from the use of three-dimensional simulation and are considered the gold standard in determining the position of the condylar head. Plain radiographs and the orthopantomogram are only useful as screening tools in intra-articular fractures since CT scanning is the only approach that provides precise imaging of the small fractured segments [14]. Thus, a CT scan is very useful for diagnosis and treatment planning of condylar fractures of the mandible.

The indications for conservative versus surgical management and surgical approach to be used depends on certain factors. When visualized in a CT scan, the condylar height can either be preserved or shortened depending on the location of the fracture line. The fracture line always runs obliquely from superolateral to inferomedial and depending on the position of the fracture line, the condylar height can be either maintained (type A) or shortened (type B). The indication for operative treatment is a type B intra-articular fracture with shortening of the condyle, whereas type A fractures which preserve condylar height are usually managed conservatively. Another important indication for open fixation includes fractures that are not communited, as the fractured segments will be too small to provide proper support for fixation [15].

The modified retromandibular approach was found to be adequate in exposing the fracture site and allowing open reduction and internal fixation but making it technically difficult. The small incision size and the height (higher level) of the intra-articular condylar head fracture make it a difficult task to reach and reduce the segments. The main reason for considering this approach as one of the choices in the management of diacapitular fractures is the lower risk of facial nerve injury as well as the most conservative of the condylar approaches in the context of soft tissue handling [16]. In the modified retromandibular approach, the area in the middle of the buccal and marginal mandibular branch of the facial nerve is utilized for dissection, thereby ensuring the protection of the facial nerve and preventing additional morbidity. The resultant scar is inconspicuous and satisfactory to the patient as it is almost always concealed in the retromandibular shadow.

## Conclusions

Treatment of fracture of the condylar head of the mandible has always been a debatable topic and nowadays surgeons are opting for surgery rather than conservative management. Open reduction and internal fixation lead to the earlier return of form and function of the temporomandibular joint, decreased facial asymmetry, and it prevents temporomandibular joint ankylosis and occlusal discrepancies. But there is a risk of damage to the facial nerve, the occurrence of facial scarring, and Frey’s syndrome. Although the modified retromandibular approach provides a satisfying and quick exposure of the condylar head, it is technically challenging and has the risk of damaging the marginal mandibular and facial nerves. However, the modified retromandibular approach will be useful in accessing the temporomandibular joint condyle from the articular fossa superiorly to the gonion inferiorly with sufficient exposure to the diacapitular fractures while giving an inconspicuous scar and should be considered an excellent and safer alternative to the preauricular approach. Thus, the modified retromandibular approach can be used in the surgical management of condylar head, condylar neck, and subcondylar fractures of the mandible, although a larger sample size would justify the utility of this modified technique over the conventional one.

## References

[1] Chen M, Yang C, He D, Zhang S, Jiang B (2010). Soft tissue reduction during open treatment of intracapsular condylar fracture of the temporomandibular joint: our institution's experience. J Oral Maxillofac Surg.

[2] Ellis E 3rd, Dean J (1993). Rigid fixation of mandibular condyle fractures. Oral Surg Oral Med Oral Pathol.

[3] Kermer Ch, Undt G, Rasse M (1998). Surgical reduction and fixation of intracapsular condylar fractures. A follow up study. Int J Oral Maxillofac Surg.

[4] Ellis E 3rd (1998). Complications of mandibular condyle fractures. Int J Oral Maxillofac Surg.

[5] Newman L (1998). A clinical evaluation of the long-term outcome of patients treated for bilateral fracture of the mandibular condyles. Br J Oral Maxillofac Surg.

[6] Iwai T, Yajima Y, Matsui Y, Tohnai I (2013). Computer-assisted preoperative simulation for screw fixation of fractures of the condylar head. Br J Oral Maxillofac Surg.

[7] Abdel-Galil K, Loukota R (2008). Fixation of comminuted diacapitular fractures of the mandibular condyle with ultrasound-activated resorbable pins. Br J Oral Maxillofac Surg.

[8] Wang WH, Deng JY, Zhu J, Li M, Xia B, Xu B (2013). Computer-assisted virtual technology in intracapsular condylar fracture with two resorbable long-screws. Br J Oral Maxillofac Surg.

[9] He D, Yang C, Chen M, Jiang B, Wang B (2009). Intracapsular condylar fracture of the mandible: our classification and open treatment experience. J Oral Maxillofac Surg.

[10] Hlawitschka M, Loukota R, Eckelt U (2005). Functional and radiological results of open and closed treatment of intracapsular (diacapitular) condylar fractures of the mandible. Int J Oral Maxillofac Surg.

[11] He D, Yang C, Chen M, Bin J, Zhang X, Qiu Y (2010). Modified preauricular approach and rigid internal fixation for intracapsular condyle fracture of the mandible. J Oral Maxillofac Surg.

[12] Vesnaver A (2008). Open reduction and internal fixation of intra-articular fractures of the mandibular condyle: our first experiences. J Oral Maxillofac Surg.

[13] Arcuri F, Brucoli M, Benech A (2012). Analysis of the retroauricular transmeatal approach: a novel transfacial access to the mandibular skeleton. Br J Oral Maxillofac Surg.

[14] Benech A, Arcuri F, Baragiotta N, Nicolotti M, Brucoli M (2011). Retroauricular transmeatal approach to manage mandibular condylar head fractures. J Craniofac Surg.

[15] Hlawitschka M, Eckelt U (2002). Assessment of patients treated for intracapsular fractures of the mandibular condyle by closed techniques. J Oral Maxillofac Surg.

[16] Kshirsagar R, Singh V, Pawar S, Shah R (2015). Retromandibular approach in the management of condylar fractures by open reduction and internal fixation a prospective study. Natl J Maxillofac Surg.

